# Feasibility, Effectiveness, and Mechanisms of a Brief Mindfulness- and Compassion-Based Program to Reduce Stress in University Students: A Pilot Randomized Controlled Trial

**DOI:** 10.3390/ijerph19010154

**Published:** 2021-12-23

**Authors:** David Martínez-Rubio, Jaime Navarrete, Jesus Montero-Marin

**Affiliations:** 1 Psicoforma, Integral Psychology Center, C/Maestro Clavé, 3, 2°, 3a, 46001 Valencia, Spain; david@psicoforma.es; 2 Excellence Research Network PROMOSAM (PSI2014-56303-REDT), 28029 Madrid, Spain; 3 Department of Psychology, Faculty of Health Sciences, Universidad Europea de Valencia, 46010 Valencia, Spain; 4 Institut de Recerca Sant Joan de Déu, 08950 Esplugues de Llobregat, Spain; 5 Teaching, Research & Innovation Unit, Parc Sanitari Sant Joan de Déu, 08830 Sant Boi de Llobregat, Spain; 6 Department of Psychiatry, Warneford Hospital, University of Oxford, Oxford OX3 7JX, UK; jesus.monteromarin@psych.ox.ac.uk

**Keywords:** mindfulness, self-compassion, university, student, mental health, stress

## Abstract

The mental health of university students is a public health concern, as psychopathology has significantly risen among this population. Mindfulness-based programs may support their mental health, though more research is needed. We used a two-armed pilot randomized controlled trial to study the feasibility, preliminary effectiveness, and potential mechanisms of a brief 6-week instructor-led mindfulness- and compassion-based program (MCBP for University Life) on perceived stress and psychological distress. Thirty undergraduate psychology students participated (15 in the intervention group, and 15 as wait-list controls). Those in the intervention arm engaged well with the course and formal at-home practice, attending at least five sessions and meditating between 4–6 days per week. Significant improvements in perceived stress, psychological distress, mindfulness skills, decentering, self-compassion, and experiential avoidance were found at the end of the intervention, while the wait-list group remained unchanged. There were significant differences between the two groups in those variables at post-test, favoring the intervention arm with major effects. Reductions in stress were mediated by improvements in mindfulness skills, decentering, and self-compassion; meanwhile reductions in psychological distress were mediated by improvements in decentering. These results suggest that this intervention might be feasible and effective for university students, but more high-quality research is needed.

## 1. Introduction

It is apparent that the mental health of university undergraduate students is a worldwide concern. A recent international survey of almost 14,000 college students showed that a substantial proportion of them (around 35%) met the diagnostic criteria of a DSM disorder [1]. In this line, a recent study found a similar percentage of pooled depression prevalence (around 25%) among university undergraduate students (*n* = 13,790), and pointed out the individual (e.g., identifying as female), interpersonal (e.g., poor social support), and systemic (e.g., academic pressures) risk factors that determine their mental health problems [2]. Furthermore, the COVID-19 pandemic has increased their risk of developing a mental illness even more, as shown in a recent cross-national prevalence study (*n* = 1,441,828). A pooled depression, anxiety, and sleep disturbance prevalence of around 30% was reported [3]. Overall, psychological distress is currently considered a focal mental health problem among university students [4,5]. This is partly due to the association of psychological distress with academic underperformance (e.g., course non-completion, failure to fulfill academic obligations, and exam performance) and other problematic health behaviors, such as substance use behaviors and suicide [6].

It has been observed that only a small proportion of university undergraduate students are receiving adequate psychological treatment [1]. However, the efficacy of several psychological interventions for this population has been studied, and there are different approaches that are currently available. Among them, mindfulness-based programs (MBPs) seem to be promising [7,8]. Specifically, Mindfulness-Based Stress Reduction (MBSR; [9]) and Mindfulness-Based Cognitive Therapy (MBCT; [10]) are the two MBPs with the most robust evidence base [11].

A systematic review and meta-analysis of randomized controlled trials have shown that MBPs might be effective by significantly improving psychological distress, depression, anxiety, well-being, rumination, and mindfulness skills among university students [7]. Mindfulness is a psychological construct that involves the ability to self-regulate attention to the events of the present moment while an accepting, open, and curious attitude is adopted toward one’s experience [12]. Thus, the purpose of MBPs is to cultivate this trait through meditation practice [13,14]. It seems that mindfulness skills, repetitive negative thinking, self-compassion, reactivity, and psychological flexibility might act as potential mechanisms of MBSR and MBCT [15]. However, MBSR, MBCT, and most of their derivations demand high commitment to the program sessions (generally eight weekly 2 h sessions), and at-home practice (45 min daily practice), which does not appeal to university students and usually results in high attrition rates [16]. For that reason, some studies have tried to make MBPs more feasible and accessible, and thus improve their adherence rates.

Though there is evidence of the effectiveness of MBPs on improving mental ill-health outcomes of university students [7], more methodological trials are needed to firmly recommend the use of MBPs to universities, and specific attention needs to be paid to implementation issues [17]. Specifically, Dawson et al. [7] suggest that the methodological deficiencies that need to be addressed include participant and outcome blinding, the randomization process, and incomplete outcome data. Moreover, the specific active components and mechanisms of change of MBPs remain unclear [7]. Some studies that tested MBPs in university students found that mindfulness skills and resilience might be potential mediators of improvements in psychological distress [18,19]. However, the emerging theoretical literature about the pathways of change of MBPs also points out the potential role of related variables such as decentering (i.e., the ability to observe thoughts and feelings in a detached manner), self-compassion (i.e., the desire to alleviate one’s own suffering), and psychological flexibility (i.e., the ability to act following personal values when experiencing negative internal circumstances), as mechanisms of action [15,20]. In this regard, experiential avoidance (the opposite of psychological flexibility) has been found to be a significant predictor of burnout among psychology and nursing undergraduate students [21]. Finally, compassion is implicitly taught in MBSR and MBCT, although these programs might enhance their effects on this variable by including specific content and compassion-based meditations [22]. Compassion-based meditations are generative contemplative practices that seek to mitigate sensitivity to suffering and strengthen the motivation to cope with it [23,24]. Specifically, it has been suggested that integrating training that explicitly focuses on intra- and interpersonal dimensions (i.e., mindfulness and compassion, respectively) might have greater effects on outcomes [22].

The essential features of MBSR and MBCT are described in order to inform adaptions to different settings [13], and the following recommendations are provided for that purpose [25]: researchers should reflect on the need for adaptation, the aim of the adaptation should be articulated, followed by the development of approaches for carrying it out, and the importance of performing feasibility studies and pilot-controlled trials to inform the design of adequate and more powerful randomized controlled trials (RCTs).

In view of the above, university students might benefit from mindfulness training and thus reduce their academic distress. However, the required commitment to practice and the length of ‘first-generation’ MBPs (e.g., MBSR or MBCT) may discourage their participation in those programs. Therefore, an MBP for university students should aim to increase retention rates by shortening the duration of the program or reducing at-home practice. For instance, Demarzo et al. [26] compared the effectiveness of 4- vs. 8-session MBPs in undergraduate students and showed that both interventions had similar effects. In addition, Berghoff et al. [27] showed that 10 min 2 week daily practice was as effective as 20 min daily practice in reducing perceived stress and increasing mindfulness skills. Furthermore, Modrego-Alarcón et al. [19] showed that virtual reality exposure enhanced retention rates and session attendance within an MBP for university students, and the MBP was effective in reducing stress even when shortening the duration of the classic mindfulness practices in benefit of the introduction of practice with virtual reality.

In this context, and using a pilot RCT, the present study explored the feasibility and preliminary effectiveness of a mindfulness- and compassion-based program, an adapted version of MBCT, for university students (MCBP for University Life) in improving perceived stress and psychological distress symptoms. In addition, the following four potential mechanisms of change were also independently explored in relation to these main outcomes: mindfulness skills, decentering, self-compassion, and experiential avoidance.

## 2. Materials and Methods

### 2.1. Participants

A total of 30 participants took part in this pilot study. All were new to meditation and meditation training. Table 1 shows the characteristics of the study participants.

### 2.2. Procedure

**Figure 1:**
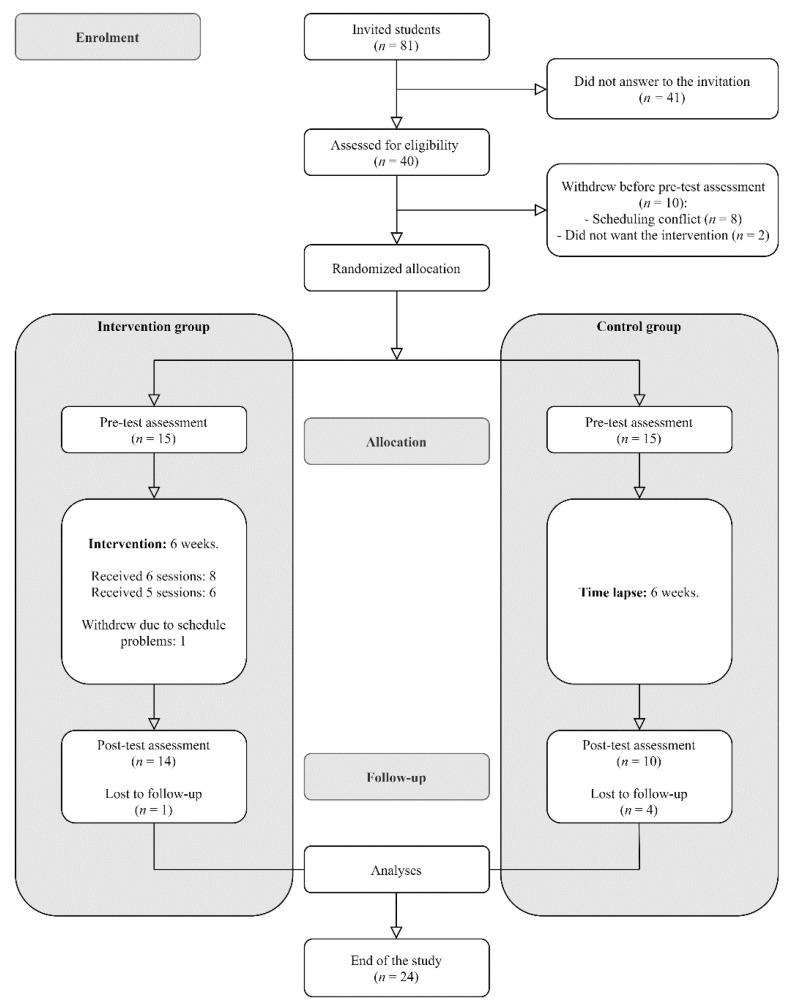
Flow of participants through the trial.

The study used a two-armed pilot RCT design. It was approved by the Institutional Review Board (Ethics Committee) of the University of Valencia (H1522866829276, 12 April 2018). The sample was recruited from the final year course of the Psychology Degree (Faculty of Psychology, Catholic University of Valencia, Spain). All students from this course were invited via email to voluntarily participate in a study about the effectiveness of a 6 week group-based MBP on stress prevention among university students. Moreover, an informative meeting was organized to explain the characteristics of the study. Those who agreed to participate gave their freely informed written consent. The week before the intervention began, they also completed a pen-and-paper survey with demographic questions and the questionnaires below. The intervention was implemented between May and June 2018. Participants had to be final-year undergraduate students over the age of 18, able to attend all sessions, and fluent in written and spoken Spanish. Participants with a current psychiatric diagnosis, undergoing psychological treatment, or showing substance use or abuse were excluded. The students who answered the invitation email were assessed for eligibility (see Figure 1 for more details) and randomly allocated to receive the brief MBP (intervention group) or to wait for 6 weeks to receive the MBP intervention (control group). At the end of the last session, participants completed the post-intervention survey.

### 2.3. Mindfulness- and Compassion-Based Program: MCBP for University Life

The MCBP we describe here for the first time is an adaptation of MBCT [10] and includes 6 in-person weekly 90 min sessions. A mindfulness teacher who was certified by the Oxford Mindfulness Centre (DM-R) with 8 years of experience teaching mindfulness and compassion groups designed the adaptation and taught the groups. The instructor also had foundational training in Compassion-Focused Therapy [28], Cognitive-Based Compassion Training [29,30], and Mindful Self-Compassion [31]. Overall, the two main new features of this adaptation are the smaller number of sessions (six sessions in total), and the incorporation of more explicit compassion training. The development of this adaptation was the result of a community-based participatory research process to meet the needs of university students, with the aim of tailoring MBCT to senior university students. To achieve this, we endeavored to maintain a balance between the original content and exercises, but also improve their feasibility mainly by reducing the time that practitioners would devote to the training in order to facilitate adherence to the program.

Within the framework of intervention development [32], the present program arose from the instructor’s experience in teaching mindfulness groups, especially in a university educational context. In addition, the demands made by university students after several workshops and informative meetings were considered, thus empowering public engagement and participation. In those meetings, students were asked to generate ideas for the new intervention in order to know the stakeholders’ views and to facilitate the feasibility, acceptability, and engagement with the new intervention. Those demands were mainly focused on shorter programs (less than 8 weeks), shorter sessions (90 min), shorter practices (15 min or less), and more emphasis was placed on training through informal practice, but also on the inclusion of creative sessions with audiovisual material (such as “the upside-down bicycle” or “the fly and the samurai”), and on the reinforcement of interpersonal dimensions, which were enhanced by including more explicit work on compassion content and practices. Thus, based on previous literature and the demands of university students, we concluded that the most important implementation issues were the time commitment and the duration of the intervention.

The program sessions included: (1) formal practices, (2) enquiry of the experience during the practice, which was usually related to the content or theme of the session, (3) review of the practice at home (except in the first session), and (4) short theoretical explanations on the theme of each session. From the first session, participants were invited to adopt an attitude of curiosity, kindness, and acceptance of whatever came up during the formal and informal practices, both in the session and throughout the week. This was presented as the central mechanism of self-discovery. These attitudes were developed and enriched throughout the program through meditation practices, as the program progressed, and were interwoven with the theoretical contents of the sessions. In addition, there was a strong emphasis on the usefulness of mindfulness practice, both formal and especially informal, as the only true vehicle for personal change. In this regard, the time allocated to formal at-home practice within the program was 9 h, with formal practice sessions of 15 min. Overall, this program is mainly characterized by short practice sessions and highlights the importance of informal practice as a way of integrating mindfulness into daily life. In relation to compassion practice, this program included from the 2nd session elements such as gratitude in the form of a 5-finger gratitude exercise, which was maintained until the end of the program, and the daily gratefulness task in the third session. Finally, the fifth session was dedicated explicitly to a theoretical and practical way to kindness and compassion.

Table 2 shows a summary of the content and meditation practices for each session (see supplementary materials Table S1 for a detailed presentation). The main themes included the following: (1) introduction to mindfulness (theory and practice), (2) reflection on the main mechanisms of action and obstacles to practice, (3) breathing and body, (4) how to relate to thoughts and emotions, (5) introduction to loving kindness and compassion, and (6) mindfulness for life.

### 2.4. Measures

#### 2.4.1. Sociodemographic Information

Participants provided information on their gender (female, male), age, marital status (single, committed relationship), perceived parental support (insufficient, good, very good), perceived social support (yes/no question), and current psychopharmacological treatment (yes/no question). Moreover, participants were asked whether they had a chronic disease, if they had previous meditation experience or had previously participated in an MBP or stress-management program.

#### 2.4.2. Feasibility

Study enrolment, acceptance of randomization, session attendance, frequency of formal at-home practice, and attrition rate were recorded in order to evaluate feasibility.

#### 2.4.3. Main Outcomes

Perceived Stress Scale (PSS; [33]). The PSS is a 14-item questionnaire used to measure to what extent respondents view their life situations as being stressful during the last month. The validated Spanish version was used [34]. Items were rated on a 5-point Likert-type scale (from 0 = “never” to 4 = “very often”). A total score (possible range: 0–56) was calculated by reversing the positive items and then adding up all of them; higher scores indicate a higher perceived stress level. Internal consistency scores in the present study were good (pre-test: α = 0.83; post-test: α = 0.86).

General Health Questionnaire (GHQ-12; [35]). The GHQ-12 is a 12-item measure to assess psychiatric strain during recent weeks. The validated Spanish version was used [36]. Items are scored on a 4-point Likert-type scale (which ranges from 0 to 3), and all scores are added to give a total score ranging from 0 to 36. Higher scores indicate a higher level of psychopathology. Internal consistency values in the present study were found to be appropriate (pre-test: α = 0.88; post-test: α = 0.92).

#### 2.4.4. Mechanistic Variables

Five Facets of Mindfulness Questionnaire—Short Form (FFMQ-SF; [37]). The FFMQ-SF is a 15-item instrument used to measure the five facets of the tendency to be mindful in daily life: Observing, Describing, Acting with Awareness, Non-judging of Inner Experience, and Non-reacting to Inner Experience. The validated Spanish version was used [38]. Items are measured on a 5-point Likert-type scale (from 1 = “never or very rarely true” to 5 = “very often or always true”) and can be added to give a total scale score, ranging from 15 to 75. Higher scores indicate higher mindfulness skills. Internal consistency in the present study was found to be good (pre-test: α = 0.80; post-test: α = 0.79).

Experiences Questionnaire—Decentering (EQ; [39]). The EQ-Decentering is an 11-item measure assessing decentering, i.e., the ability to observe thoughts and feelings in a detached manner. The validated Spanish version was used [40]. Items are scored on a 5-point Likert-type scale (from 1 = “never” to 5 = “always”) and all scores are added to form a total score that ranges from 11 to 55. Higher scores indicate a higher level of decentering. Internal consistency was good in the present study (pre-test: α = 0.80; post-test: α = 0.88).

Self-Compassion Scale—Short Form (SCS-SF; [41]). The SCS-SF is a 12-item self-report measure assessing self-compassion. Items are scored on a 5-point Likert-type scale (from 1 = “almost never” to 5 = “almost always”). To compute a total score, negative items are reversed and then the mean of all items is calculated [42]. Higher scores indicate a higher self-compassion level. The validated Spanish version was used [43]. Internal consistency was good in the present study (pre-test: α = 0.80; post-test: α = 0.90).

Acceptance and Action Questionnaire-II (AAQ-II; [44]). The AAQ-II comprises 7 items based on a 7-point Likert-type scale (from 1 = “never true” to 7 = “always true”). It was designed to assess experiential avoidance (as the opposite of psychological flexibility), with higher scores indicating a higher level of avoidance. Scores for all items are added up to give a total score ranging from 7 to 49. The validated Spanish version was used [45]. Internal consistency in the present study was found to be good (pre-test: α = 0.90; post-test: α = 0.91).

### 2.5. Data Analysis

Baseline socio-demographic characteristics and feasibility were descriptively analyzed (mean, standard deviation; frequency, percentage). The scales’ internal consistency was established by calculating Cronbach’s alpha coefficient, with values higher than 0.70 being considered adequate [46]. The variables were then checked for unexpected missing data (percentages), normality (kurtosis ranging from −2 to +2 and skewness from −7 to +7, Shapiro–Wilk’s statistic with *p* > 0.05, histograms, and Q–Q plots), homogeneity of the groups in the pre-test scores, and outliers to ensure there were no major violations of main assumptions.

Paired-sample t-tests were conducted to evaluate the intra-group impact of the intervention/time lapse on students’ scores on PSS (perceived stress), GHQ-12 (psychopathology), FFMQ-SF (mindfulness), EQ (decentering), SCS-SF (self-compassion), and AAQ-II (experiential avoidance) for each group. Cohen’s d effect size was used, with cut-off values of 0.2, 0.5, and 0.8 for small, medium, and large effect sizes, respectively [47]. Then, differences between groups’ post-test scores were explored by using analysis of covariance (ANCOVA). The independent variable was the group (MBP, waitlist). Pre-test scores were treated as covariates to control for baseline differences between groups. The partial eta squared effect size was used, with cut-off values of 0.01, 0.06, and 0.14 for small, medium, and large effect sizes, respectively [47].

Change scores were calculated by subtracting pre-test from post-test scores of PSS, GHQ-12, FFMQ-SF, EQ, SCS-SF, and AAQII. Pearson’s correlations were then used to explore the linear relation between change scores of main outcomes (perceived stress and psychological strain) and the potential mediators of change (mindfulness skills, self-compassion, experiential avoidance, and decentering). The effect size guidelines for interpreting small, medium, and large correlations were 0.10 to 0.29, 0.30 to 0.49, and 0.50 to 1, respectively [47].

Finally, simple mediation analysis using ordinary least squares path analysis was carried out following the methodology described by Hayes [48]. We entered group (MBP, waitlist) as the independent variable, the change scores of the mechanistic variables as possible mediators, and the change scores for perceived stress or psychological strain as the dependent variable. Path a denoted the relation between the group and the change scores of the mechanistic variables; the association between change scores of the mechanistic variables and main outcomes was denoted by path b. The confidence interval (CI) for the indirect effect (ab) was a percentile bootstrap 95% interval based on 5000 samples. CIs that did not contain the zero value indicated a significant indirect effect. The multiple determination coefficient effect size was used, with cut-off values of 0.14, 0.39, and 0.59 for small, medium, and large effect sizes, respectively [49].

All the analyses were performed using IBM SPSS Statistics version 26, and JASP version 0.14.1. Given the exploratory nature of the current study, we used an overall alpha level of 0.05 and did not correct for multiple testing [50].

## 3. Results

### 3.1. Feasibility

From all the students of the final year course (*n* = 81) who were invited to take part in the study, 40 answered the invitation email and were assessed for eligibility (see Figure 1 for more details), of whom 30 were randomly allocated. All participants accepted the condition to which they were randomly assigned (15 per condition). Overall, five participants withdrew from the study prior to completing follow-up metrics (see Figure 1 for more details, and supplementary materials Table S2 for sociodemographic characteristics of the final sample). Of the MBP participants, all of them attended session 1, 93% session 2, 86% session 3, 93% session 4, 86% session 5, and 100% session 6. Overall, 93% of the MBP group attended at least five of the six sessions, and 53% attended all sessions. Participants reported engaging in formal at-home practice between 4–6 days per week.

### 3.2. Effectiveness of the MBP

Perceived stress levels at baseline were moderate based on the data from the validation study [34]. The pre-test levels of psychological strain in both conditions were also high according to normative data for the Spanish population as a whole [36]. Table 3 shows that there was a statistically significant decrease in total scores of PSS (*p* < 0.001; *d* = 1.20), and GHQ-12 (*p* < 0.001; *d* = 1.35) from pre-test to post-test assessment in the MBP group, with large effect sizes for both main outcomes. Moreover, statistically significant increases were found for FFMQ-SF (*p* < 0.001), SCS-SF (*p* < 0.001), and EQ (*p* < 0.001) scores, and decreases for AAQ-II (*p* < 0.001) scores between assessment moments. Cohen’s d indicated large effect sizes in all the process variables, ranging from 0.79 to 1.8. In contrast, the control group remained unchanged overall.

After adjusting for pre-test scores, there was a statistically significant difference between the two groups on post-intervention scores on the PSS (*p* < 0.001; *η_p_^2^* = 0.43), GHQ-12 (*p* = 0.001; *η_p_^2^* = 0.40), FFMQ-SF (*p* < 0.001; *η_p_^2^* = 0.47), SCS-SF (*p* < 0.001; *η_p_^2^* = 0.41) AAQ-II (*p* < 0.001; *η_p_^2^* = 0.47), and EQ (*p* < 0.001; *η_p_^2^* = 0.48), with large effect sizes.

### 3.3. Correlations between Variables and Change Scores

Correlations between pre-test scores of all variables and between mediator change scores are available as supplementary materials (Tables S3 and S4, respectively). As can be seen in Table 4, reductions in PSS scores were strongly associated with improvements in FFMQ-SF, SCS-SF, and EQ, as well as reductions in AAQ-II scores. Moreover, there was a strong correlation between GHQ-12 and FFMQ-SF, SCS-SF, and EQ change scores, with larger reductions in GHQ-12 being associated with larger improvements in FFMQ-SF, SCS-SF, and EQ (see Table 4 for more details).

### 3.4. Mediating Role of Mindfulness Skills, Decentering, Self-Compassion, and Experiential Avoidance

**Figure 2:**
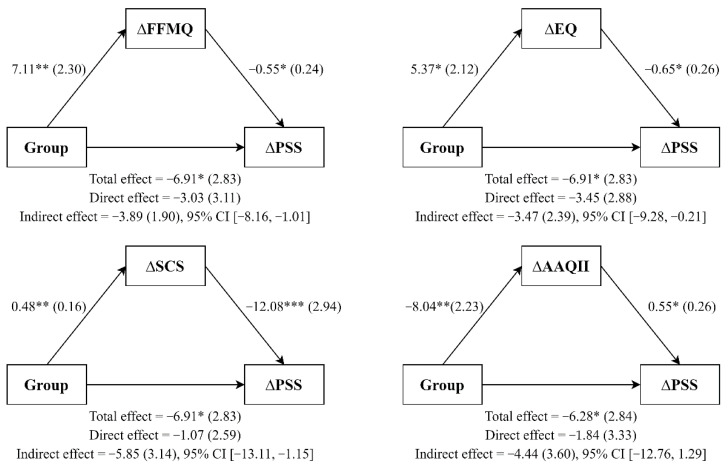
Simple mediation analyses. Note. All coefficients represent unstandardized regression coefficients (standard errors in brackets). * *p* < 0.05; ** *p* < 0.01; *** *p* < 0.001.

**Figure 3:**
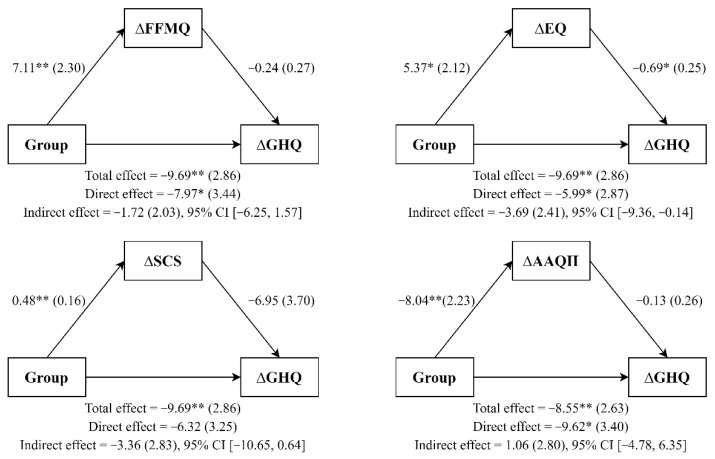
Simple mediation analyses. Note. All coefficients represent unstandardized regression coefficients (standard errors in parenthesis). * *p* < 0.05; ** *p* < 0.01.

Simple mediation analysis (see Figure 2 and Figure 3) showed that MBP participants (vs. WL participants) reported significant improvements in mindfulness skills (a = 7.11; *p* = 0.005), and that these improvements predicted changes in perceived stress (b = −0.55; *p* = 0.03) but did not do so in psychological strain (b = −0.24; *p* = 0.374). The confidence intervals for the interaction effects on perceived stress did not cross zero, which indicated a possible mediating effect of mindfulness skills on perceived stress (ab = −3.89; 95% CI [−8.16, −1.01]). This mediating model explained a figure of 37% (R squared = 0.37) of perceived stress. However, mindfulness improvements did not mediate the group effect on psychological strain (ab = −1.72; 95% CI [−6.25, 1.57]; R squared = 0.36). Furthermore, MBP participants (vs. WL participants) reported significant improvements in decentering (a = 5.37, *p* = 0.019). In turn, changes in decentering predicted reductions in perceived stress (b = −0.65; *p* = 0.019) and psychological strain (b = −0.69; *p* = 0.013). The 95% bias-corrected bootstrap confidence interval for the interaction effects on perceived stress (ab = −3.47; [−9.28, −0.21]) and psychological strain (ab = −3.69; [−9.36, −.14]) did not cross zero, indicating a possible mediating effect of decentering. The mediating models explained 40 and 51% of perceived stress (R squared = 0.39) and psychological strain (R squared = 0.51), respectively.

Moreover, MBP participants had higher improvements in self-compassion (a = 0.48; *p* = 0.005) than WL participants, and these improvements predicted changes in perceived stress (b = −12.08; *p* < 0.001) but not in psychological strain (b = −6.95; *p* = 0.074). On one hand, the 95% bias-corrected bootstrap confidence interval for the interaction effects revealed a mediating effect of self-compassion on perceived stress (ab = −5,85; [−13.11, −1.15]); this model accounted for 56% of the variance (R squared = 0.56). On the other hand, improvements in self-compassion did not mediate the group effect on psychological strain (ab = −3.36; [−1.65, 0.64]; R squared = 0.44). Finally, MBP participants (vs. WL participants) reported significant improvements in experiential avoidance (a = −8.04; *p* = 0.002). These improvements predicted changes in perceived stress (b = 0.55; *p* = 0.044) but not in psychological strain (b = −0.13; *p* = 0.619). The confidence interval indicated that experiential avoidance mediated neither perceived stress (ab = −4.44; [−12.76, 1.29]; R squared = 0.34) nor psychological strain (ab = −1.06; [−4.78, 6.35]; R squared = 0.34).

## 4. Discussion

This pilot RCT studied the feasibility, preliminary effectiveness, and potential mediators of the “MCBP for University Life” course in a sample of senior university students. It was a shortened version of the MBCT that explicitly included compassion content [22], and sought to overcome the feasibility and acceptability limitations of “first-generation” MBPs studies observed with university students [7,16]. The results of the present study showed that university students engaged well with the course, and significantly improved their perceived stress, psychological strain, mindfulness skills, decentering, self-compassion, and experiential avoidance. No improvements were revealed for the WL control group during the same period of time, who remained unchanged overall. In addition, the effect of the intervention on perceived stress seemed to be mediated by the improvements in terms of mindfulness skills, decentering, and self-compassion. Additionally, we have seen that changes in decentering could mediate the improvements on psychological strain, suggesting some potential pathways of change of the MBP used. The relevance of the study consists in the fact that it presents an adaptation tailored to the expressed wants and needs of the students (i.e., shorter and fewer sessions). For that purpose, frequency of formal practice was reduced while maintaining the essence of MBCT and its effectiveness. Instead, informal practices were emphasized, and compassionate practices were included.

Engagement with the course was high, with 93% of the sample attending at least five sessions of the MBP and all participants meditating between 4 and 6 days per week at home. Furthermore, 80% of participants (93% of the intervention group and 67% of the control group) answered the post-test assessments. Previous studies have suggested, with regard to the engagement of university students with standard MBPs, that the higher the length of the courses and the required commitment with practice, the higher the attrition rates [16]. In this vein, attrition rates higher than 30% have been reported in MBSR studies with university student samples (e.g., [51]). Thus, the main purpose of the adaptation, i.e., to make the program more feasible and available to university students, was achieved.

As we have observed, the MBP had a significant effect on decreasing perceived stress and psychological strain at post-intervention, which is consistent with the findings from systematic reviews and meta-analyses within university student samples [7,52]. It should be noted that the present results were obtained by comparing the effectiveness of the intervention with a passive control group, as is the case with most previous studies on MBPs for university students [7]. Moreover, baseline levels of perceived stress and psychological strain were moderate to high in the entire sample, which might partially explain the large effect of the intervention, as they had considerable room for improvement. Similarly, a four-session, 2 h per week group adaptation of MBCT for medical students was found to be effective on perceived stress and general psychological distress [53]. Moreover, Galante et al. [17] reported significant reductions in psychological distress, thus supporting the preliminary efficacy of an 8 week MBP adapted for university students. More recently, these authors have confirmed the effectiveness of this MBP on psychological distress over the long term [54]. Regarding the duration of the intervention and formal practice, the most frequently researched MBPs for university students had a frequency of 8 weeks and encouraged participants to formally practice at least 20 min a day, i.e., 16 h devoted to at-home formal practice [7]. Accordingly, our results showed that the effects of the MCBP for University Life might be comparable to those of previous programs, even encouraging participants to engage in a shorter duration of formal practice, i.e., 15 min a day, 9 h in total.

In addition, consistent with previous research [15,18,19,55], in general, the more participants increased their mindfulness skills, decentering, and self-compassion, the more they experienced a reduction in perceived stress and psychological strain. Interestingly, the potential mediator role of self-compassion observed might extend the rather modest evidence of this variable as a mechanism of change [15]. In line with Brito-Pons et al. [22], our results suggest that explicitly teaching self-compassion skills might increase the effect of MBPs by incorporating this potential mechanism in the therapeutic process with more strength. Regarding experiential avoidance, our results did not support its mediator role despite the theoretical rationale and preliminary evidence of being a potential mechanism of change in MBPs (e.g., [56]). This may be due to the fact that this study focuses more on aspects related to compassion than most MBPs, thus lessening the time invested on psychological flexibility explicit contents.

The main strength of this study is the RCT design, which allowed for an exploration of the effectiveness of the “MCBP for University Life”. It will also allow a powered RCT to be designed to definitively confirm those exploratory benefits. However, this study also has several limitations. First, individual at-home practice, acceptability, and credibility were not recorded. Second, the small sample size precludes making definitive conclusions about the effectiveness of the mindfulness program and potential underlying variables. Furthermore, mediation analyses might have been underpowered because of the small sample size. Third, the sample consisted of psychology students, which might have positively biased the results of the intervention since they might be more predisposed to accept psychological interventions. Fourth, there were no follow-up assessments to adequately evaluate either causal chains of mechanistic effects, or the lasting effect of improvements, and only self-report measures were used, with their subsequent limitations regarding possible social desirability trends.

Thus, a future adequately powered study should compare the effectiveness of the “MCBP for University Life” in a larger trial including follow-up assessments, objective measures to complement questionnaires, adherence to meditation practice records, and acceptability and credibility assessments. Moreover, the effectiveness of the MBP should be compared to an active control group to achieve a better representation of its true effects.

## 5. Conclusions

Besides the exploratory nature of the present study, the adaptation of “MCBP for University Life” showed preliminary effectiveness on students’ stress and mental health. The results of this study make it possible to design and conduct a powered and larger trial. The described potential mediator role of mindfulness skills, decentering, and self-compassion might help therapists to improve outcomes by reinforcing the program content that is related to these constructs. Specifically, these results justify a greater insistence on compassion content within the MBP adapted to university students.

## Figures and Tables

**Table 1:** Characteristics of study participants.

Baseline Characteristic	MBP Condition (*n* = 15)		WL Condition (*n* = 15)		Full Sample (*n* = 30)	
	M	SD	M	SD	M	SD
Age	22.08	3.65	22.5	4.64	22.29	4.17
	***n***	**%**	***n***	**%**	***n***	**%**
Gender						
Female	11	73	14	93	25	83
Male	4	27	1	7	5	17
Marital status						
Single	8	53	9	60	17	57
Committed relationship	7	47	6	40	13	43
Perceived parental support						
Insufficient	2	13	0	0	2	7
Good	8	53	8	53	16	53
Very good	5	34	7	47	12	40
Perceived social support ^a^	12	80	10	67	22	73
Previous participation in stress management programs ^a^	1	6	1	6	2	7
Having a chronic disease ^a^	2	13	3	20	5	17
Previous medication ^a^	2	13	1	6	3	10

Note. ^a^ Shows the number and percentage of participants answering ‘yes’ to this question.

**Table 2:** Sessions of the mindfulness- and compassion-based program for university life.

Session	Program Topic	Meditations and Practices
Session 1	Introduction to the basics	Mindful raisin-eating meditation The 3-step breathing space
Session 2	Obstacles to practice	Body Scan (breath–body) Conscious movements
Session 3	The breath and the body	Mindfulness of breathing focusing on the belly Mindfulness of nose-focused breathing
Session 4	Thoughts and emotions	The samurai and the fly (video) Attentive listening 50/50
Session 5	Kindness and compassion	Sounds and thoughts Compassion (and self-compassion)
Session 6	Mindfulness for life	Group reflection on key learning points Guidelines to keep practicing independently in our daily lives

Note. Presented exercises are examples of those meditations and practices taught in each session.

**Table 3:** Means, standard deviations, paired samples *t*-test, and ANCOVA comparing primary and secondary outcomes.

Measurement	MCBP Condition					WL Condition					ANCOVA		
	Pre-Test	Post-Test	t (13)	*p*	*d*	Pre-Test	Post-Test	t (9)	*p*	*d*	F (1, 21)	*p*	*η_p_^2^*
PSS	27.86 (8.05)	18.14 (4.35)	4.49	<0.001	1.20	30.30 (4.92)	27.50 (6.72)	2.01	0.076	0.63	15.82	<0.001	0.43
GHQ-12	16.21 (6.53)	5.93 (5.53)	5.06	<0.001	1.35	14.29 (6.29)	14.00 (6.33)	0.33	0.749	0.10	14.19	0.001	0.40
FFMQ-SF	48.36 (7.96)	56.07 (4.27)	−5.04	<0.001	−1.35	46.57 (8.83)	47.00 (9.29)	−0.36	0.729	−0.11	18.25	<0.001	0.47
EQ	36.29 (6.64)	44.36 (4.13)	−5.99	<0.001	−1.60	32.36 (5.42)	33.80 (5.57)	−1.63	0.137	−0.52	19.22	<0.001	0.48
SCS-SF	3.19 (0.51)	3.86 (0.34)	−6.75	<0.001	−1.80	2.53 (0.65)	2.71 (0.75)	−1.49	0.170	−0.47	14.77	<0.001	0.41
AAQII	24.21 (8.53)	16.08 (6.34)	4.44	<1.001	1.23	24.64 (1.10)	27.00 (9.85)	−0.40	0.698	−0.13	37.06	<0.001	0.47

Note. Standard deviations are shown in brackets. PSQ = Perceived Stress Questionnaire; PSS = Perceived Stress Scale; GHQ-12 = General Health Questionnaire; FFMQ-SF = Five Facets of Mindfulness Questionnaire—Short-Form; EQ = Experiences Questionnaire; SCS-SF = Self-Compassion Scale—Short Form; AAQII = Acceptance and Action Questionnaire.

**Table 4:** Pearson’s correlations for change scores among primary and mechanistic outcomes.

	∆ PSS	∆ GHQ-12
∆ FFMQ-SF	−0.58 **	−0.45 *
∆ EQ	−0.60 **	−0.64 **
∆ SCS-SF	−0.75 **	−0.58 **
∆ AAQII	0.58 **	0.29

Note. *n* = 24. Primary outcomes: PSS = Perceived Stress Scale; GHQ-12 = General Health Questionnaire. Mechanistic outcomes: FFMQ-SF = Five Facets of Mindfulness Questionnaire—Short-Form; EQ = Experiences Questionnaire; SCS-SF = Self-Compassion Scale—Short Form; AAQ-II = Acceptance and Action Questionnaire. * *p* < 0.05. ** *p* < 0.01.

## Data Availability

Data available on request from the authors.

## Supplementary Materials

The following are available online at https://www.mdpi.com/article/10.3390/ijerph19010154/s1, Table S1: Specific content of MCBP for University Life sessions, Table S2: Sociodemographic Characteristics of Final Sample Participants, Table S3: Pearson’s correlations for pre-test scores, Table S4: Pearson’s correlations between mediators change scores. D.M.-R., J.M.-M. and J.N.
