# Supplementary material for: Feasibility, Effectiveness, and Mechanisms of a Brief Mindfulness- and Compassion-Based Program to Reduce Stress in University Students: A Pilot Randomized Controlled Trial
Source: Int J Environ Res Public Health. 2021 Dec 23;19(1):154. doi: 10.3390/ijerph19010154 (PMC8750204; doi:10.3390/ijerph19010154)
Supplement: Supplementary file 1 [file ijerph-19-00154-s001.zip › ijerph-1502263-supplementary.pdf]

TABLE S1. Specific content of MBCT for University Life sessions.

	Structure and rationale	Exercises and practices during the session	Homework
1	**Introduction to the basics**<br>Class Orientation (Welcome, Format, Intentions).<br>What is Mindfulness. How to practice it What do we need?<br>Mindfulness vs Autopilot. Formal and informal practice<br>Basic concepts and attitudes	• Observing our inner experience and motivation. Why are we here?<br>• Raisin mindful eating meditation<br>• The 3-step breathing space (3SBS)<br>• Mindfulness of breathing	• Mindfulness of breathing<br>• Mindful eating<br>• 3SBS<br>• Practic log
2	**Obstacles to practice**<br><br>Reflection on the main obstacles for practice<br>Mechanisms of action and benefits of practice.<br>The brains of people who practice<br>Two modalities: Doing vs being<br>Primary and secondary suffering<br>Breathing into the body.	• 3SBS<br>• Body Scan (Breath-body)<br>• Conscious movements (standing and lying down)<br>• Video (bike upside down)	• Body Scan<br>• Mindfulnn movements and stretches<br>• 3SBS<br>• Mindful eating<br>• Mindful Shower (shower paying attention to the different senses)<br>• Mindful Sip (pay attention to the first sip of any drink we drink throughout the day, even for a few seconds)<br>• Gratitude (5 finger gratitude exercise)
3	**The breath and the body**<br><br>Breathing and body.<br>Relationship of respiration and states of mind.<br>Why breathing and utilities. Breathing practices<br>Expand body work	• Mindfulness of breathing focusing into the belly<br>• Mindfulness of nose focused breathing.<br>• Breathing counting<br>• Mindful movement and walking<br>• 3SBS	• Mindful movements and mindful waking<br>• 3SBS<br>• Breathing into the abdomen or counting breaths<br>• Mindful Shower and Mindful Sip<br>• Gratitude exercise<br>• Daily gratefulness
4	**Thoughts and emotions**<br><br>Thoughts and biases. How we interpret the world<br>How to relate to thoughts. Acceptance / resignation<br>Thought patterns<br>Thoughts and emotions. Feeling in the body	• 3SBS (new versión)<br>• The samurai and the fly (video)<br>• What is a thought like?<br>• Breathing abdomen and tip of the nose<br>• Sounds and thoughts / feelings (MBCT practice)<br>• Attentive listening 50/50	• Mindful movements and. Mindful Displacement<br>• 3SBS<br>• Mindful Shower<br>• Attentive listening 50/50<br>• Gratitude<br>• Sounds and thoughts
5	**Kindness and compassion**<br><br>What is and what is not compassion<br>Self pity and kindness<br>Biological bases of Compassion (CFT- Systems)<br>The self-care system<br>How can I take better care of myself?	• Sounds and thoughts<br>• Compassion<br>• Self Compassion<br>• Nutritious and strenuous activities exercise	• 3SBS<br>• Attentive listening 50/50<br>• Mindful eating.<br>• Mindful Shower<br>• Mindful Displacement<br>• Self kindness and compassion<br>• Random act of kindness
6	**Mindfulness for life**<br><br>Compassion and adherence to practice.<br>Last reflection and clearing doubts about compassion.<br>Acquiring guidelines to sustain our practice.	• Kindness and compassion mediation<br>• Group reflection on key learning points.<br>• Guidelines to keep practicing independently in our daily lives<br>• Collective reflection and conclusions<br>• Mindfulness of gratitude.	

TABLE S2
Sociodemographic Characteristics of Final Sample Participants

Baseline characteristic	**MBP condition**		**WL condition**		**Full sample**	
	n	%	*n*	%	*N*	%
**Gender**						
Female	10	71	10	100	20	83
Male	4	29	0	0	4	17
**Marital status**						
Single	8	57	5	50	13	54
Committed relationship	6	43	5	50	11	46
**Perceived parental support**						
Insufficient	2	14	0	0	2	8
Good	7	50	3	30	10	42
Very good	5	36	7	70	12	50
**Perceived social support** [a]	12	86	8	80	20	83
**Previous participation in stress management programs** [a]	1	7	1	10	2	8
**Having a chronic disease** [a]	2	14	3	30	5	21
**Previous medication** [a]	2	14	1	10	3	13

[a] Reflects the number and percentage of participants answering 'yes' to this question.

TABLE S3

Pearson's correlations for pre-test scores

Variable		1	2	3	4	5	6
1. PSS	Pearson's r	—					
	p-value	—					
2. GHQ-12	Pearson's r	0.656	—				
	p-value	< .001	—				
3. FFMQ-SF	Pearson's r	-0.518	-0.273	—			
	p-value	0.010	0.197	—			
4. EQ	Pearson's r	-0.524	-0.523	0.314	—		
	p-value	0.009	0.009	0.135	—		
5. SCS-SF	Pearson's r	-0.662	-0.371	0.430	0.686	—	
	p-value	< .001	0.074	0.036	< .001	—	
6. AAQII	Pearson's r	0.678	0.353	-0.406	-0.351	-0.693	—
	p-value	< .001	0.091	0.049	0.092	< .001	—

Note. FFMQ-SF = Five Facets of Mindfulness Questionnaire – Short-Form; EQ = Experiences Questionnaire; SCS - SF = Self-Compassion Scale – Short Form; AAQ-II = Acceptance and Action Questionnaire.

TABLE S4

Pearson's correlations between mediators change scores

Variable		1	2	3	4
1. Δ FFMQ-SF	Pearson's r	—			
	p-value	—			
2. Δ EQ	Pearson's r	0.332	—		
	p-value	0.113	—		
3. Δ SCS-SF	Pearson's r	0.441	0.628	—	
	p-value	0.031	0.001	—	
4. Δ AAQII	Pearson's r	-0.475	-0.285	-0.540	—
	p-value	0.022	0.187	0.008	—

Note. FFMQ-SF = Five Facets of Mindfulness Questionnaire – Short-Form; EQ = Experiences Questionnaire; SCS - SF = Self-Compassion Scale – Short Form; AAQ-II = Acceptance and Action Questionnaire.
